# Dysregulation of neutrophil death in sepsis

**DOI:** 10.3389/fimmu.2022.963955

**Published:** 2022-08-18

**Authors:** Cheng-long Zhu, Yi Wang, Qiang Liu, Hui-ru Li, Chang-meng Yu, Peng Li, Xiao-ming Deng, Jia-feng Wang

**Affiliations:** Faculty of Anesthesiology, Changhai Hospital, The Naval Medical University, Shanghai, China

**Keywords:** neutrophil, cell death, sepsis, apoptosis, NETs, necroptosis, pyroptosis, autophagy

## Abstract

Sepsis is a prevalent disease that has alarmingly high mortality rates and, for several survivors, long-term morbidity. The modern definition of sepsis is an aberrant host response to infection followed by a life-threatening organ dysfunction. Sepsis has a complicated pathophysiology and involves multiple immune and non-immune mediators. It is now believed that in the initial stages of sepsis, excessive immune system activation and cascading inflammation are usually accompanied by immunosuppression. During the pathophysiology of severe sepsis, neutrophils are crucial. Recent researches have demonstrated a clear link between the process of neutrophil cell death and the emergence of organ dysfunction in sepsis. During sepsis, spontaneous apoptosis of neutrophils is inhibited and neutrophils may undergo some other types of cell death. In this review, we describe various types of neutrophil cell death, including necrosis, apoptosis, necroptosis, pyroptosis, NETosis, and autophagy, to reveal their known effects in the development and progression of sepsis. However, the exact role and mechanisms of neutrophil cell death in sepsis have not been fully elucidated, and this remains a major challenge for future neutrophil research. We hope that this review will provide hints for researches regarding neutrophil cell death in sepsis and provide insights for clinical practitioners.

## Introduction

Sepsis is defined as a severe, potentially lethal organ dysfunction which results from an imbalanced host response to various infection (1). Until now, the pathogenesis of sepsis has not been fully clarified, and the mortality remains alarmingly high as a result of the lack of effective therapeutic strategies. Over the past decade, research on this disease has shifted its focus from systemic inflammatory response syndrome (SIRS) towards multiple organ dysfunction syndrome (MODS), and many advances and progress have been made (2). Sepsis can be caused by various injuries (trauma, burn, infection, etc.), but inflammation and immune-mediated pathology are usual features of the illness pathophysiology (2). In many patients requiring critical care, pro- and anti-inflammatory responses are activated simultaneously and innate immunosuppression becomes an important and potentially reversible complication of sepsis (3). With the rapid progress of sepsis, the mortality rate of septic patients remains 25%-30%, and even as high as 40%-50% in septic shock (4), which still remains a major concern in critical care filed at the moment (5).

The initial inflammatory response to sepsis is usually driven by innate immune cells in the immune system, such as neutrophils, monocytes, and macrophages, which have the ability to release a number of inflammatory cytokines (6). Neutrophils have a key role in inflammatory pathophysiology and immunological dysregulation induced by sepsis and act as the host initial important line of defense to fight infections (2). Neutrophils have a powerful antimicrobial effect, but this is also the reason for a double-edged role as both an important guardian of host defenses and a harmful facilitator of tissue damage in an uncontrolled inflammatory state (7). In septic patients, dysregulated neutrophil cell death may be hazardous because it predisposes neutrophils to immune-related organ failure, resulting in reduced body defenses and increased vulnerability to hospital-acquired infections. As a result, neutrophils have evolved from potent antibacterial components to potentially harmful mediators of tissue damage and organ failure.

**Figure 1:**
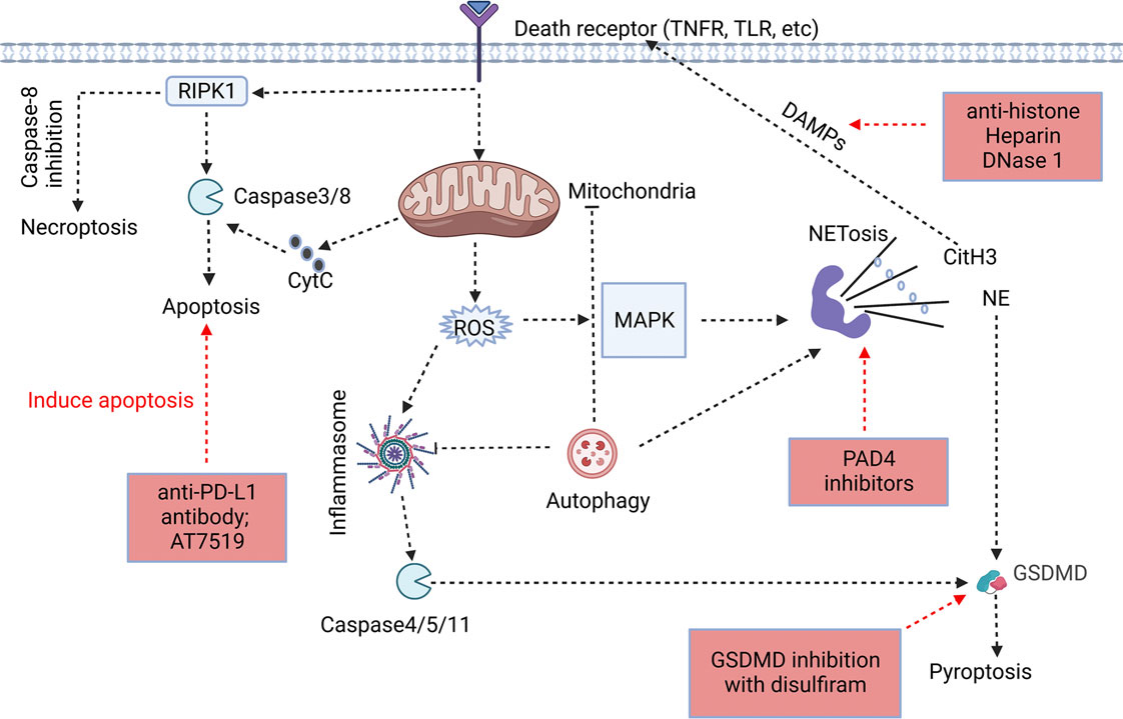
Association of different forms of programmed neutrophil cell death. The forms and specific mechanisms about neutrophil cell death in sepsis are not clear. Neutrophil apoptosis is delayed in sepsis, and necroptosis may occur when caspase-8 activity is inhibited. Neutrophils may release NETs through NETosis, and CitH3 in NETs may cause cell death as DAMPs. GSDMD, a key protein in pyroptosis, is involved in the release of NETs, and NE in NETs has the ability to cleave GSDMD to cause pyroptosis. Autophagy, as an intracellular degradation system, can recover harmful substances in cells and prevent the spread of inflammation, but it may assist in the formation of NETs despite of the ongoing controversy (32, 34–36). The process of cell death usually begins at the receptor itself. However, the signaling cascade determines the fate of neutrophils to some extent. In addition, we summarized the therapeutic potential strategies of neutrophil death for sepsis, including induction of neutrophil apoptosis, GSDMD inhibition, anti-NETosis, and anti-DAMPs (Red Boxs).

Neutrophils are inherently short-lived, with a half-life of 18-19 hours (8). Under normal circumstances, neutrophils generally die through apoptosis, a programmed death; nevertheless, if the host suffers from a severe infection, neutrophils will probably experience multiple additional types of cell death (9). And under septic insults, our group found that the spontaneous apoptosis of neutrophils was inhibited and the longevity of neutrophils would be increased (10). Despite that apoptosis is inhibited, neutrophils may undergo some other types of cell death in sepsis (necrosis, necroptosis, pyroptosis, NETosis, and autophagy, which are different from apoptosis) (11–14) (**Table 1**). The pathological stimuli or changes in the external environment will induce a shift of death type in neutrophils and leading to overwhelming inflammatory responses (9, 33). Moreover, several types of cell death may affect each other, and the same molecule may induce different types of cell death (**Figure 1**). This article reviews the significance of the neutrophil cell death in the onset and progression of sepsis and its potential therapeutic role in sepsis, which can provide us with new insight to better understand the pathogenesis of sepsis and with new strategies for the treatment of sepsis.

**Table 1:** Comparison of features in different types of cell death.

	Necrosis	Apoptosis	Necroptosis	Pyroptosis	NETosis	Autophagy
Cell membrane	Rupture of plasma membrane (15)	Plasma membrane blebbing; Rounding-up of the cell (16)	Rupture of plasma membrane (17)	Pore formation and rupture of plasma membrane (18)	Rupture of plasma membrane (19)	Lack of change (20)
Cytoplasm	Swelling of cytoplasmic organelles (15)	Retraction of pseudopods; Cell volume reduction (16)	Swelling of the cytoplasm (translucent cytoplasm) and cytoplasmic organelles (17)	Cytoplasmic swelling (translucent cytoplasm) (18)	Massive vacuolization of the cytoplasm; Breakdown of the granular membranes (19)	Accumulation of double-membraned autophagic vacuoles (20)
Nucleus	Moderate chromatin condensation (15)	Nuclear fragmentation; Chromatin condensation (16)	Moderate chromatin condensation (17)	Nuclear fragmentation; Chromatin condensation (21)	Rapid chromatin decondensation; Breakdown of the nuclear membranes (19)	Lack of chromatin condensation (20)
Cellular morphology	Increase in size and deformity (15)	Shrink (16)	Increase in size and deformity (17)	Increase in size and deformity (18)	Increase in size and deformity (19)	Produce vacuoles (20)
Biochemical features	Inhibition of caspases; Fragmentation of oligonucleosomal DNA; Decrease in ATP levels (9, 14, 15)	Activation of caspases; Δψm dissipation; Inhibition of respiratory chain (12, 22)	Death receptor signaling; Caspase inhibition; Activation of RIP1, RIP3, and MLKL; Decrease in ATP levels (17, 23)	Caspase-1/4/5/11 activation; Caspase-7 activation; Secretion of IL-1β and IL-18 (18, 24)	Activation of NADPH oxidase; Inhibition of caspase; NET release (for some instances) (19, 25, 26)	LC3-I to LC3-II translation; Substrate (ex, p62) degradation (20, 27)
Immune features	Most frequently pro-inflammatory because DAMPs are released (ex, HMGB1) (9)	Usually anti-inflammatory and immunological-silent; in certain situations, provoking an immune response as a result of DAMP exposing and releasing (ex, DNA, histone and HMGB1) (13)	Pro-inflammatory in most situations because DAMPs are released (ex, HMGB1); anti-inflammatory in other instances (13, 17)	Often pro-inflammatory due to release of IL-1β and IL-18 (18)	Pro-inflammatory because DAMPs are released (ex, histones); anti-inflammatory in other situations due to the death of bacteria, fungus, viruses, and parasites (19, 25, 28)	Anti-inflammatory due to suppression of inflammasome activation; pro-inflammatory in some situations due to mediation of non-classical cytokine release (20, 29, 30)
Change of neutrophil death in sepsis	Undefined	Downregulated (31)	Undefined	Undefined	Upregulated (28)	Upregulated (32)

## Neutrophil homeostasis

Neutrophils, a type of polymorphonuclear leukocyte, are an essential element of the body innate immune system and contain diverse “weapons” for battling infections (37). Neutrophils were previously regarded as a homogeneous population of cells with potent antimicrobial capabilities, including phagocytosis, degranulation, and production of neutrophil extracellular traps (NETs) (37, 38). However, recent studies have shown that neutrophils can be divided into several heterogeneous groups, such as low-density neutrophils (LDNs), high-density neutrophils (HDNs), and intermediate-density neutrophils (IDNs), which are important regulatory components of the immune response (38–40). In humans, neutrophils make up 50-70% of all circulating white blood cells (41). An adult produces about 100 billion neutrophils averagely every day (42). Once mobilized into the circulation, neutrophils have a relatively short half-life, between 18 and 19 hours (8). The rate of neutrophil production, storage and excretion in the bone marrow, as well as their survival and removal mechanisms in the circulation, are major factors determining the overall amount of neutrophils within the body (37). In order to accommodate to the environmental challenges, the host must constantly adjust itself to achieve a balance between production and clearance of neutrophils. As a result of their cytotoxicity, it is important to firmly establish proper development and clearance systems to guard the body against unintentional inflammatory harm. Under normal physiological conditions, neutrophils maintain a certain number mainly through apoptosis. However, it was reported that the apoptosis of peripheral blood neutrophils from sepsis patients was inhibited (31), while the release of neutrophil NET increased in the murine septic model (43), and the autophagy level of peripheral blood neutrophils in septic patients increased (32). Unfortunately, other ways of neutrophil cell death have not been well understood in sepsis. Due to the existence of different neutrophil cell death pathways, it is crucial to comprehend how they differ mechanistically in order to develop potential targeted therapies for sepsis (**Figure 1**).

## Necrosis

When neutrophils leave the peripheral circulation under noninfectious conditions, the majority of them undergo apoptosis (44), where the noxious agents are phagocytosed by macrophages. Necrosis, by contrast, is a chaotic type of cell death. As an anti-infective cell, when the body is infected with bacteria, neutrophil can phagocytose bacteria, and then necrosis occurs to form pus after the phagocytosed bacteria are dissolved by lytic enzymes (45). Toxic components such as proteolytic and oxidative producing enzymes may be released from necrotic cells during infection, causing damage to body. Necrosis is caused by unfavorable environmental conditions such as hypoxia or a lack of necessary nutrients, high temperatures, poisonous chemicals, and mechanical stress. Ca^2+^ and reactive oxygen species (ROS) are two of the most common participants in necrosis, regardless of triggering factor. Ca^2+^ levels in the cytosol rise during necrosis, causing calcium overload and the activation of proteases and phospholipases in mitochondria (46). Because of ion imbalance and membrane integrity loss, ROS can damage lipids, proteins, and DNA, causing mitochondria to become dysfunctional. Necrotic cells can release diverse danger signals called damage associated molecular patterns (DAMPs), including DNA chromatin compounds, high mobility group box 1 (HMGB1), heat shock proteins (HSPs), uric acid, and antimicrobial peptides (9), which can trigger the production of proinflammatory mediators by activating pattern recognition receptors (PRRs) (9).

Neutrophil necrosis can be caused by tumor necrosis factor (TNF)-α, which is released by activated neutrophils or other immune cells like dendritic cells and monocytes by activating nuclear factor kappa B (NF-кB) in surrounding cells (such as macrophages or fibroblasts) (47, 48). The cytoplasm of necrotic neutrophils is enlarged, and the nuclei are disorganized. The early death of neutrophils, however, may cause this shape to alter. Some neutrophils will become necrotic under the apoptotic pathway if the damage is severe enough, which is known as “secondary necrosis” (49). It has been reported that upon infection with Haemophilus influenzae, it is phagocytosed by neutrophils, which activates the secretion of IL-8 and respiratory burst. Nevertheless, instead of killing bacteria on their own, neutrophils release their granule contents into the extracellular milieu by necrosis, resulting in neutrophil infiltration that further amplifies inflammation (50). Although the necrosis of neutrophils in sepsis has not been clearly studied, theoretically, neutrophils in sepsis will also undergo necrosis when they die during resisting against microorganisms, leading to the production of pus. Neutrophil necrosis may be one of the main culprits of tissue injury and organ dysfunction in sepsis (51). But neutrophils may also undergo a series of molecular changes, leading to changes in the types of cell death in sepsis, and the actual proportion of necrotic cells remained to be determined in systemic and circulating neutrophils during sepsis.

## Apoptosis

Apoptosis is necessary for eliminating the accumulation of neutrophils in inflammatory tissues without releasing damaging cell inclusions (52). Apoptotic neutrophils are engulfed by macrophages to limit the inflammatory responses (53, 54). Phagocytes that bind and ingest apoptotic cells can induce positive immunosuppressive and anti-inflammatory responses, and phagocytosis by apoptotic cells not only inhibits the production of a diverse array of pro-inflammatory cytokines in necrotic cell-activated macrophages, but there is also transparent *in vivo* evidence that transforming growth factor (TGF)-β1, which is secreted by macrophages after ingesting apoptotic cells, has an anti-inflammatory property in the peritoneum and lung with inflammation (55). But apoptosis does have a direct pro-inflammatory effect, causing the release of IL-18 and IL-1β by caspase-1-dependent pathway (56, 57). This process can be triggered in an extrinsic or intrinsic way. The extrinsic way of apoptosis is usually triggered by the interaction of TNF-α or extracellular ligands such like Fas ligand (FasL) with particular cell surface TNF receptors (58). And the intrinsic pathway is initiated through various damaging stimuli involving the mitochondrial release of cytochrome c into the cytoplasm (59), which activates the intracellular caspases that are in charge of cleaving DNA and cytoplasmic structural proteins (60). In addition, normal adults produce 10^11^ mature neutrophils every day. Neutrophil apoptosis is an important mechanism to maintain an appropriate number of neutrophils under physiological conditions (61, 62).

One of the most consistent and profound alterations in neutrophils during sepsis is that they activate a survival program that prevents the constitutive tendency of neutrophil apoptosis after release from the bone marrow. A study showed that 50% of resting neutrophils exhibited apoptotic morphological changes after 24 hours of *in vitro* culture, while the corresponding percentage for septic neutrophils was only 5 - 10% (31). Sepsis in human is associated with a severe suppression of neutrophil apoptosis rate. Activation of the anti-apoptotic environment and the presence of anti-apoptotic neutrophils may lead to the systemic inflammatory damage and multi-organ dysfunction of sepsis, even if extended neutrophil survival may reflect an adaptive resistance to infection (31, 63). Neutrophil apoptosis is negatively correlated with the severity of sepsis in septic patients, suggesting that it may be a measure of sepsis severity inside this group (64). In acute lung injury (ALI), lung neutrophil apoptosis was significantly reduced within 24 hours after injury following ligation and puncture (CLP) or endotoxemia (10, 65). We and others found that neutrophil apoptosis was significantly reduced in patients with sepsis, and the plasma from these sufferers proved capable of preventing the apoptosis of neutrophils from the healthy controls (10, 63). These data suggested that inhibition of neutrophil apoptosis increased the possibility of tissue/organ injury in experimental and clinical settings.

**Figure 2:**
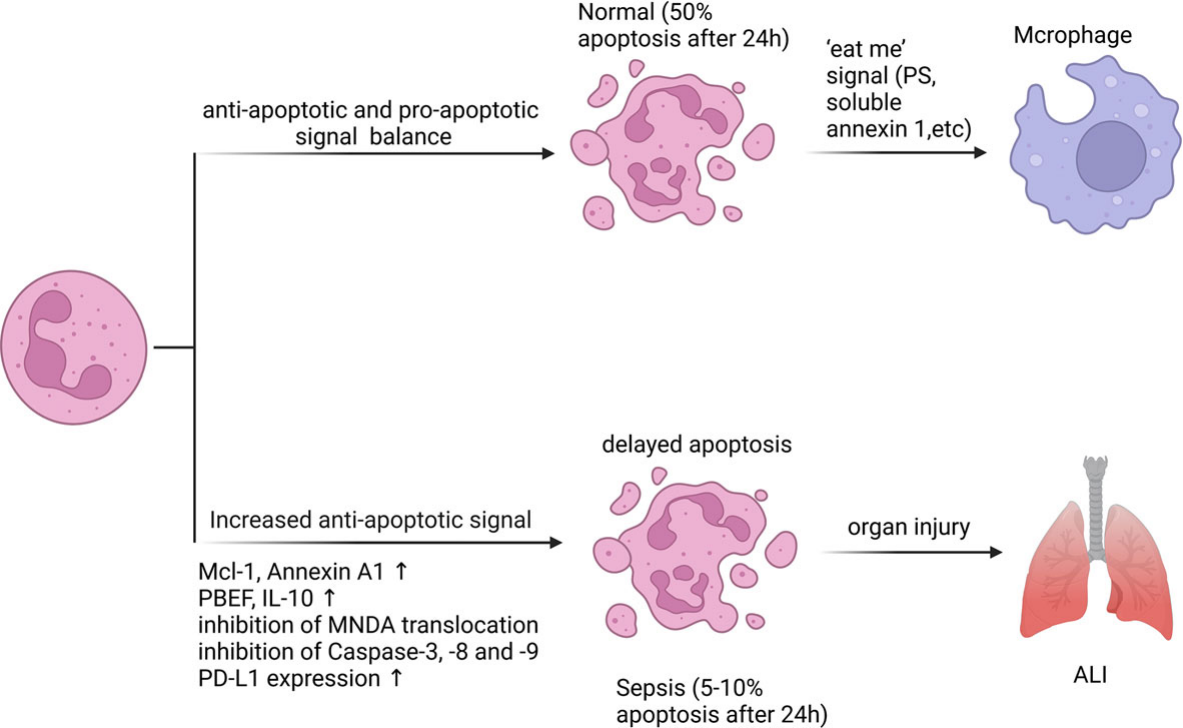
Neutrophil apoptosis is inhibited in sepsis. Apoptosis is essential for regulating the lifespan of neutrophils. In sepsis, the execution of the neutrophil death program is delayed by various stimuli. Inhibition of neutrophil apoptosis or reduction of macrophage uptake of apoptotic neutrophils can exacerbate injury to tissues or organs. 50% of resting neutrophils exhibited apoptotic morphological changes after 24 hours, while the corresponding percentage for septic neutrophils was only 5 - 10%. A variety of anti-apoptotic signals produced during sepsis include preservation of Mcl-1 and Annexin A1, release of PBEF, IL-10, inhibition of MNDA translocation from nucleus to cytoplasm, inhibition of Caspase-3, -8 and -9 activation, and increased PD-L1 expression.

During sepsis, signaling pathways that promote neutrophil survival converge to control the expression and degradation of key factors affecting apoptosis (**Figure 2**). The activation of anti-apoptotic factors appears to be the primary cause of the delayed neutrophil apoptosis (66). Although neutrophils do not specifically express B cell lymphoma-2 (Bcl-2), they are able to express B-cell lymphoma-extra large (Bcl-xL), Annexin A1, Bak, and myeloid cell leukemia-1 (MCL-1), which are anti-apoptotic participants of the Bcl-2 family and can reduce apoptosis across both internal and external pathways (67–69). In both clinical and experimental sepsis, Pre-B cell colony-enhancing factor (PBEF) (70) and IL-10 (71) are essential for the delayed neutrophil apoptosis. A study on neutrophil apoptosis in patients with sepsis showed that the mechanism of delayed apoptosis involves the activation of NF-κB, which is related to the decrease of caspase-3 and caspase-9 levels as well as the preservation of mitochondrial transmembrane potential (31). Dysregulated phosphorylation and inhibition of the catalytic activity of caspase-8 also contribute to the survival of septic neutrophils (72, 73). In addition, the cytoplasmic aggregation of myeloid nuclear differentiation antigen (MNDA) is crucial in the apoptotic process of neutrophils. It is mainly located in the nucleus, regulates the degradation of Mcl-1, and then accumulates mitochondrial function in the cytoplasm (74). One study showed that bacterial lipoprotein (BLP) inhibited mitochondrial membrane depolarization of neutrophils and subsequently reduced caspase-3 activation, eventually resulting in a considerable delay of neutrophil apoptosis (75). Adenosine 2A receptor (A2AR)-inhibited autophagy inhibits neutrophil apoptosis by blocking caspase-8, caspase-3 and polyadenosine-diphosphate-ribose polymerase (PARP) signal transduction, which determines a new anti-inflammatory effect of A2AR in regulating neutrophil survival during inflammation (76). Recently, some scholars have shown a novel method to specifically target the pro-inflammatory neutrophils apoptosis process using doxorubicin-conjugated protein nanoparticles, which may reduce neutrophils infiltration and associated inflammatory responses in sepsis-induced lung injury, and improve the survival rate of sepsis (77). Moreover, our previous study indicated that increased programmed death ligand-1 (PD-L1) expression on neutrophils from septic patients delayed neutrophil apoptosis by triggering phosphatidylinositol 3-kinase (PI3K) dependent Akt phosphorylation, and conditional knockout of PD-L1 in neutrophils attenuated lung injury and improved survival rate in septic mice with CLP (10). In general, the regulatory mechanism of neutrophil apoptosis inhibition during sepsis is complex, and new methods of regulating neutrophil apoptosis are being explored.

## Necroptosis

Both apoptotic and non-apoptotic types of cell death can occur in neutrophils, with the latter appearing to become more associated with inflammatory situations. Neutrophils can undergo a regulated kind of necrotic death known as necroptosis, which is dependent on the activity of the enzymes mixed-spectrum kinase-like (MLKL) and receptor-interacting protein kinase 3 (RIPK3) (78, 79). Necroptosis seems to be a form of programmed cell death, however unlike apoptosis, it is unable to result in DNA fragmentation or the apoptosis-like morphological changes (78, 79). Necroptosis in neutrophils can be caused by activating toll like receptors (TLRs), death receptors, intracellular DNA and RNA sensors, interferons (IFNs)-α receptor agonists, or adhesion molecules (including CD44, CD11b, CD18, and CD15) induction (78–80). In addition, several groups have reported that necroptosis in neutrophils can also be induced by phagocytosis of *Staphylococcus aureus* (81, 82), or exposure to monosodium urate (MSU) crystals (83, 84). Recent research by Schauer et al. showed that MSU crystals could cause NET aggregation (85). And Desai et al. found deficiency of RIPK3 inhibited MSU crystal-induced NET formation, confirming the involvement of neutrophil necroptosis along NET release (83).

Necroptosis has been recognized as a crucial process of inflammation-mediated cell death. Inhibition of necroptosis can attenuate sepsis-induced lung injury (86), kidney injury (87), and hepatic injury (88). The therapeutic importance of RIPK kinase inhibition during sepsis is highlighted by the fact that RIPK3 deletion defends against sepsis models (89, 90). Inhibition of RIPK3 or RIPK1 reduces systemic inflammation and reduces organ damage in septic neonatal mice (86, 91). A clinical study showed that in patients with sepsis, the expression of RIPK3 and MLKL connected with plasma HMGB1 levels, which were related to the severity and mortality of the condition (92). Additionally, the research conducted by Chen et al. showed that RIPK3 mediated necroptosis could collaborate with GSDMD induced pyroptosis (see below) in the process of sepsis, enriching inflammatory signaling pathways and enhancing tissue damage (93), which may imply that multiple cell deaths occur during sepsis and together contribute to the development of sepsis.

**Figure 3:**
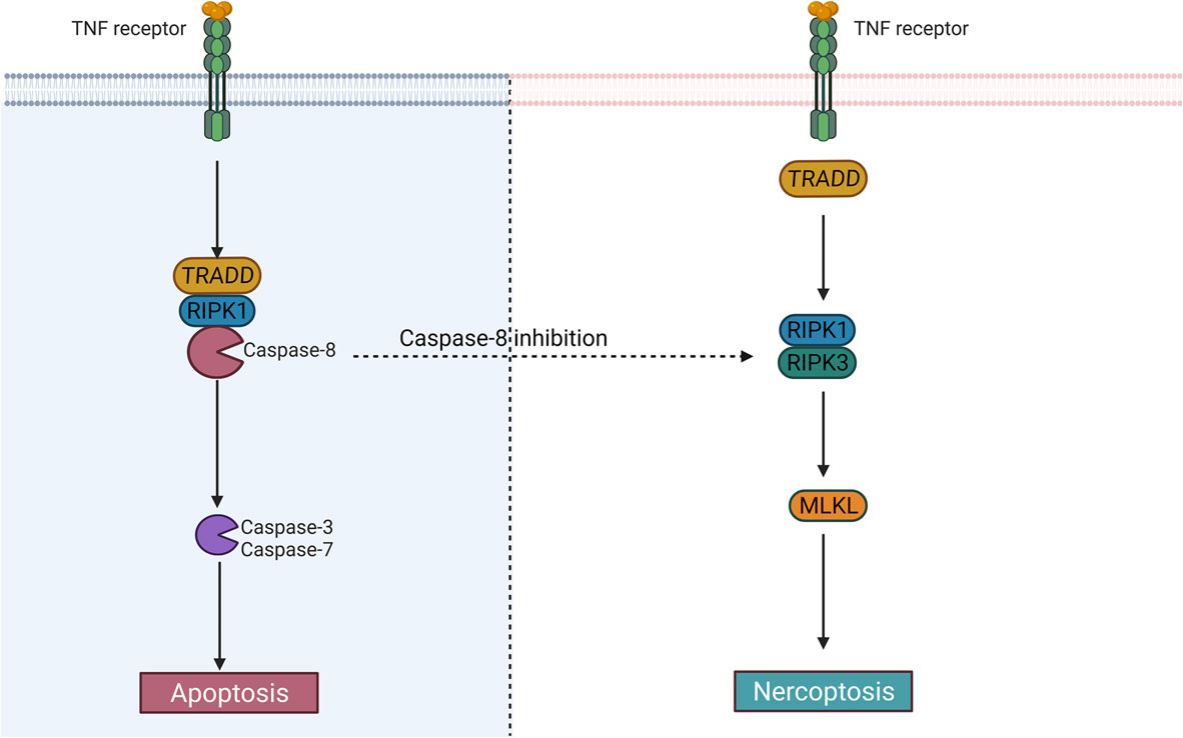
The difference between apoptosis and necroptosis. Caspase-8 largely determines whether cells undergo an apoptotic or necrotic program. After activation of caspase-8, the cells may undergo apoptosis; When it is inhibited, the cells may become necroptotic.

Whether cells die *via* apoptosis or necroptosis may depend on caspase-8 activity (**Figure 3**), but the exact molecular mechanism is currently unclear (79). Although septic neutrophil apoptosis is inhibited and the catalytic activity of caspase-8 may be suppressed (73), the significance of neutrophil necroptosis in the mechanism of human sepsis is unclear because signaling of necroptosis, apoptosis, and pyroptosis (PANoptosis, a coordinated cell death pathway) can overlap and the precise mechanisms that determine whether neutrophil dies through apoptosis or necroptosis or pyroptosis remain yet unknown (40, 79).

## Pyroptosis

**Figure 4:**
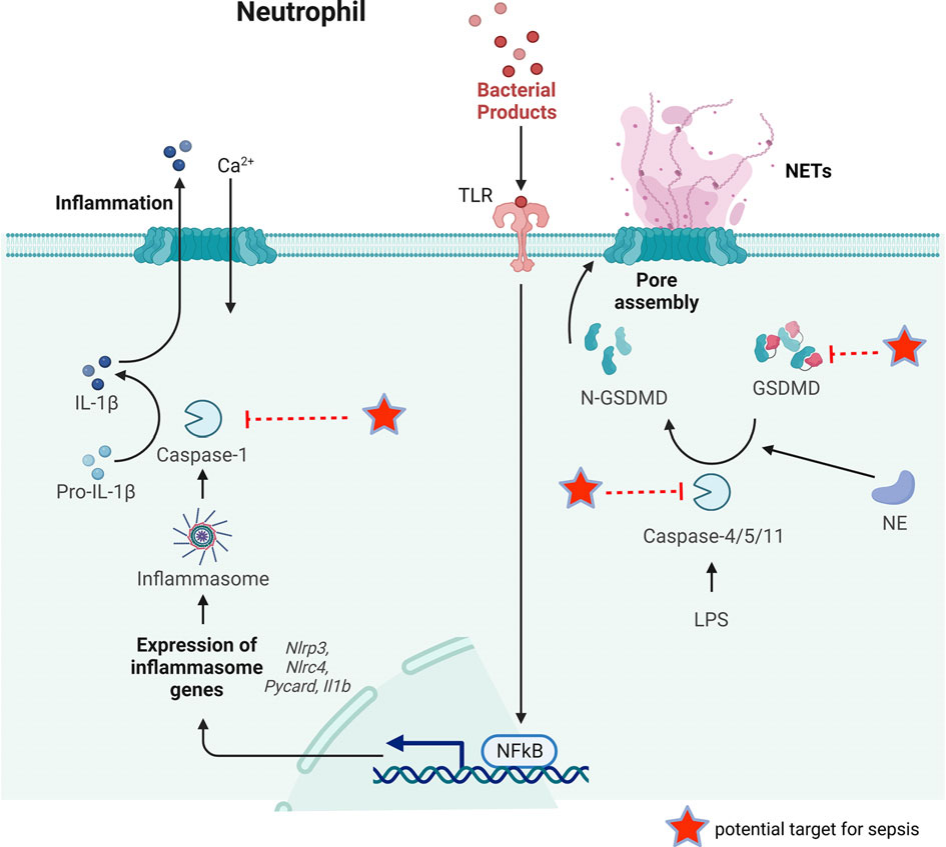
The key process of neutrophil pyroptosis in sepsis. In the classical pathway, pathogens, bacteria and other signals can recognize the intracellular NLR family and activate casepase-1 by forming inflammasome. Capsase-1 can cleave pro-IL-1β to IL-1β inducing inflammation. Whereas in the non-classical pathway, the participation of inflammasome is not necessary for the activation of caspase-4/5/11. Capsase-4/5/11 can cleave GSDMD to N-GSDMD and promote NET release. GSDMD can also be cleaved by NE to promote NETs release. Some possible targets for reducing NETs release during sepsis are pointed out.

Pyroptosis is regarded as a programmed cell death linked to inflammatory condition that was initially discovered in macrophages and related disorders (94). At present, most reports on cell pyroptosis are concentrated on monocytes and macrophages (95). Although there is no exact report that neutrophils can present pyroptosis in a specific environment, a significant set of reports confirm that neutrophils may also show pyroptosis (96, 97). Pyroptosis has long been considered as the caspase-1-mediated cell death as a response to some bacterial attacks (98). However, many recent studies have identified gasdermin D (GSDMD), a substrate of the classical pathway mediated by caspase-1 and the non-classical pathway mediated by caspase- 4/5/11, as the executor of pyroptosis (**Figure 4**) (99, 100). In the classical pathway, the intracellular Nod-like receptor (NLR) family is recognized by certain signals such as bacteria, pathogens, which allows them to activate casepase-1 by connecting to pro-caspase-1 *via* the adaptor protein ASC. Whereas in the non-classical pathway, the participation of ASC is not required for activation of caspase-4/5/11. A substantial gasdermin family with novel membrane pore-forming activity is represented by GSDMD. Therefore, gasdermin-mediated programmed necrosis was introduced as a new definition for pyroptosis.

The pyroptosis eliminates the intracellular bacterial replication ecotone, and the immune factors released *via* pyroptosis can activate effector cells that kill pathogens, thus exerting a protective effect (101). However, the release of pro-inflammatory cytokine IL-1β appears to exacerbate the pro-inflammatory response and the spread of local tissue damage, which has been confirmed in a murine model of melioidosis (102, 103). It was reported that IL-1β can be released by pyroptosis *via* the classical pathway, which lowers the murine survival rates (104). However, in 2014, Chen et al. clarified that activation of the NLRC4 inflammasome in neutrophils during acute *Salmonella* infection triggered IL-1β maturation, but upon activation of the NLRC4 inflammasome, neutrophils did not go through pyroptosis like macrophages do (105). Contrary to other myeloid cells, neutrophils are difficult to undergo pyroptosis triggered by caspase-1, even if the generation of IL-1 β is sustained (105, 106). Furthermore, N-GSDMD does not reside in the plasma membrane (PM) or improve PM permeability, which are essential for pyroptosis, despite being necessary for IL-1β secretion in NLRP3-activated mouse and human neutrophils, according to Karmakar et al. (107). This tolerance to classical pyroptosis mediated by caspase-1 extends neutrophil life, allowing neutrophils to continue IL-1β production at the infection site without jeopardizing critical non-inflammatory somatic non-dependent antimicrobial effector functions, including phagocytosis and killing of pathogens at the site of inflammation (105, 106). Notably, Kremserova et al. demonstrated that after phagocytosis of community-associated methicillin-resistant *Staphylococcus aureus*, the human neutrophil released IL-1β according to a formerly unknown mechanism that was reliant on serine proteases and RIPK3, but not on typical NLRP3 inflammatory vesicles as well as activation of caspase-1 (108). Therefore, whether neutrophils die of classical pyroptosis has always been the focus of debate.

Because of the limited expression of caspase-1 and the traditional inflammasome signal transduction adaptor protein ASC in neutrophils, the non-classical pyroptosis mediated by caspase-4/5/11 is particularly crucial in the activation of neutrophil GSDMD (106). According to Kumari et al., the activation of caspase-11 in neutrophils was essential and required for lipopolysaccharide-induced death during sepsis, however, it had little effect on other cells, like intestinal epithelial cells (109). Silva et al. found that neutrophil caspase-11 deficiency reduced GSDMD cleavage and NET release, which confirmed that GSDMD activity was located in the downstream of initial caspase-11 activation (28). The deletion of caspase-11 does not completely reduce the activation of GSDMD, suggesting that other components are needed for the activation of GSDMD (28). Sollberg et al. and Kambara et al. both demonstrated that GSDMD was also cleaved by neutrophil elastase (NE) (110, 111). The existence of cytoplasmic LPS can be sensed by the non-canonical inflammasome, transfection of LPS into the cytoplasm of murine neutrophils induced pyroptosis (106). Interestingly, the pyroptosis cells discharge their chromatin as well. The involvement of caspase-11 in NET release was also recently confirmed in septic neutrophils (28, 106). These discoveries suggest an association between pyroptosis and NET formation in neutrophils.

In sepsis, the main proteins in neutrophil pyroptosis (caspase-1/11, GSDMD) play a crucial role. Gentile et al. found that endogenous caspase-1 caused lower survival and increased inflammatory cytokine levels in polymicrobial sepsis, and that caspase-1/11 ablation improved neutrophil phagocytosis (112). Caspase-11 was required for neutrophil pyroptosis and was required to eliminate *B. thailandensis* infection, according to Kovacs et al. (113). In murine sepsis models, gene deletion or antagonism of GSDMD with disulfiram reduced NET release and improved organ function, leading to enhanced survival in septic mice (28). Furthermore, caspase-11-knockout mice, rather than caspase-1-knockout mice, can reduce neutrophil NET release and protect against CLP-induced sepsis (28). Chen et al. found that RIPK3^-/-^, GSDMD^-/-^, or RIPK3^-/-^GSDMD^-/-^ prevented cell death induced by inflammatory cytokines and HMGB1, implying that blocking both necroptosis and pyroptosis reduced tissue injury and led to improved bacterial clearance in septic mice (93). There is a lot of evidence suggesting neutrophil pyroptosis may be involved in sepsis (103). The main mechanism of pyroptosis has been discovered recently, but on the other hand, the potential benefit of neutrophil pyroptosis in the treatment of sepsis, has received insufficient attention.

## Neutrophil extracellular trap formation (NETosis)

**Figure 5:**
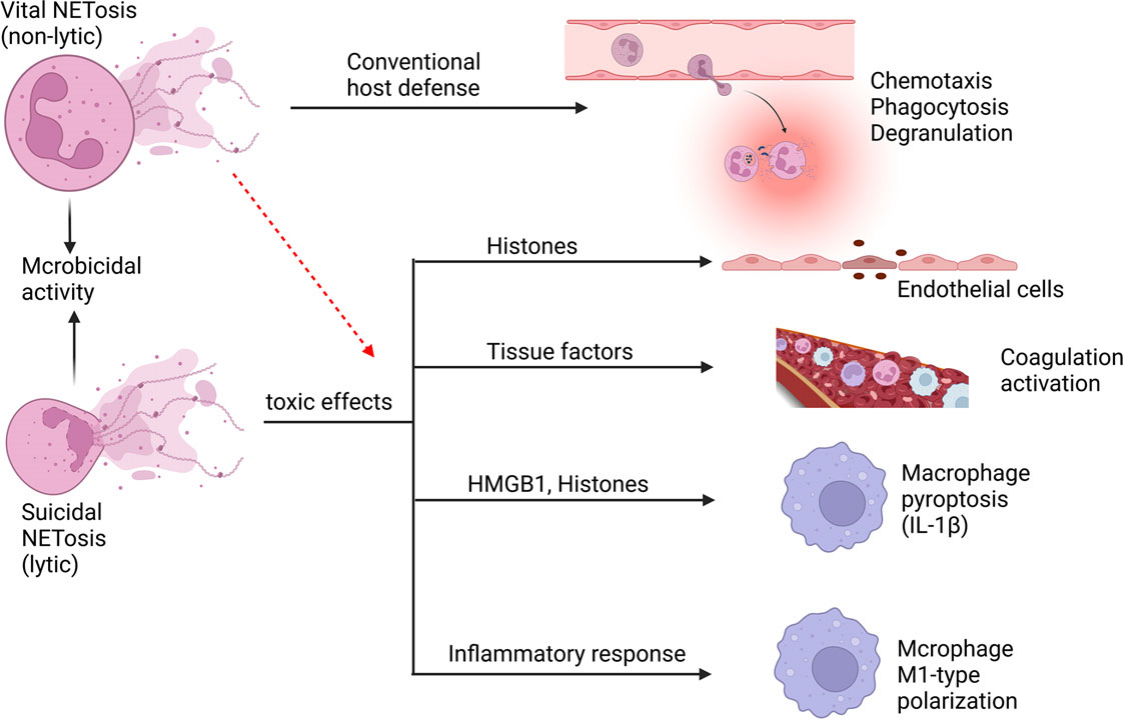
Neutrophil NETosis in sepsis. Suicidal NETosis and vital NETosis are two types of NETosis that differ mainly in whether or not the neutrophils cleave. The primary function of vital NETosis is to offer extracellular antimicrobial effects, ensuring that neutrophils remain mobile and phagocytic, but too much NET release also cause injury. Suicidal NETosis primarily causes some damage to the body, but also provides microbicidal activity. NETs and their components, such as histones and DNA, are cytotoxic and have the ability to damage endothelial cells. Tissue factors can be created and released during the creation of NETs, triggering the coagulation cascade while also boosting platelet activation, resulting in platelet aggregation and ultimately inducing thrombosis formation. NETs may induce macrophage pyroptosis, which will aggravate the inflammatory responses during sepsis. In addition, NETs may induce M1-type polarization of macrophages of lung tissue, which increases lung inflammation and lung injury.

Neutrophils have the ability to release NETs to trigger first-line innate immune responses to different stimuli, such as fungi, bacteria, and protozoa. In 2004, Brinkmann et al. reported the involvement of NETs in infection control for the first time (114). However, this is not exclusive to neutrophils and has been demonstrated in all granulocytes treated by IL-5, C5a, IFN- γ, or GM-CSF (115). NETs are a kind of reticular chromatin-based structure, which is introduced into the extracellular context to help clear pathogens, however, they are related to excessive inflammatory response, autoimmunity enhancement and vascular thrombosis as well (116). NETs contain proteins from azurophilic granules, such as myeloperoxidase (MPO), NE, and cathepsin G, as well as nuclear proteins such as histones H1, H2A, H2B, H3 and H4. In addition, proteins from secondary and tertiary particles, such as lactoferrin and gelatinase, can also be found in NETs (117). The release of NETs occurs mainly through a process of cell death called NETosis (116). NETosis involves multiple sequential steps, including the neutrophil nuclear and cytoplasmic granule membranes being disrupted, chromatin becoming relaxed, chromatin interacting with granule proteins, and chromatin being released from neutrophils (54, 118). Suicidal NETosis and vital NETosis are two types of NETosis that differ mainly in whether or not the neutrophils cleave (119) (**Figure 5**). Suicidal NETosis is a cellular suicidal behavior during which neutrophil cell membranes rupture and lose conventional functions of living neutrophils, including phagocytosis, leukocyte recruitment, and chemotaxis. In contrast, vital NETosis primarily provides extracellular antimicrobial effects, ensuring that neutrophils remain mobile and phagocytic (119). However, Yousef et al. indicated that the term NETosis was misleading, which did not apply to characterize a special type of neutrophil cell death because it denotes a combination of NET formation and DNA release after necrosis (120). Neutrophil necrosis with releasing DNA and NET formation are suggested to be viewed as two independent events and cell necrosis may be not a necessary condition for NET formation (120). Neutrophils must be stimulated with PMA for at least 2 hours to cause “NETosis”, while for NET formation, only a low concentration of PMA stimulation for 10 minutes is sufficient (114). Neutrophil necroptosis allows the release of a nuclear cloud, and RIPK3 KO neutrophils, although unable to undergo necroptosis, rapidly form NETs (121), and the mechanisms required for this are certainly different. This argues that NET formation, necrosis, and necroptosis are totally different processes. PMA induces NETosis through a NOX-dependent pathway. But some recent findings suggested that NOX-independent NET showed an increased ability to stimulate endothelial cells to play a greater role in sepsis compared with NOX-dependent NET (122). Furthermore, the issue of whether NETs contain chromosomal DNA remains a point of discussion (123). On one hand, NETs have been shown to be generally composed of mtDNA (124–127), on the other hand, regarding NETs of nuclear origin, there have been recent papers that have studied the role of chromatin in NETosis (128, 129). For example, some have shown that chromatin DNA sustains oxidative damage during NETosis, and that nuclear DNA repair is necessary for NETosis to take place (128). This supports the notion that chromatin can be the source of NETs. Transcription has also been shown to be necessary for NETosis (129). PMA-stimulated neutrophils may initially develop NETs containing mtDNA and then undergo follow-up necrosis *in vitro*. It has been demonstrated that nuclear DNA produced by dying neutrophils after PMA stimulation remains a DNA cloud instead of DNA fibers (130). Although it may be the method of DNA release measurement, the different time points of measurement, and the different stimuli received by neutrophils that contribute to this discrepancy, we are still cautious that neutrophil necrosis may not be a requirement for NET formation.

In addition to their beneficial effects against foreign invasion, NETs also have a number of deleterious implications on host tissues during sepsis (43, 131, 132). In sepsis, endothelial cell (EC) activation is viewed as an important part, in which NETs and their components play an important role (132). NETs can synergize with IL-1α and cathepsin G to facilitate the activation of ECs and enhance the formation of thrombosis, which may amplify and spread the thrombus related EC dysfunction caused by epidermal erosion (133). During sepsis, NETs can promote platelets aggregation by promoting sinusoidal endothelial cell separation, which causes liver dysfunction (134). Triggering receptor expressed on myeloid cells 1 (TREM-1) has the ability to promote NET release from neutrophils during sepsis, which can stimulate ECs and damage vascular reactivity, and TREM-1 inhibition suppressed these harmful effects (135). NETs also possess components that act as damage‐associated molecular patterns (DAMPs), which can contribute to endothelial damage and tissue injury (136). Protein components in NETs, especially histones, cause cytotoxicity in ECs (136–138). In septic mice, extracellular histone release leads to neutrophil infiltration, EC vacuolization, intra-alveolar hemorrhage, and vascular thrombosis (138). LPS can activate peptide arginine deiminase (PAD) and mediate NET formation *via* the PAD-NET pathway during sepsis, thereby altering pulmonary vascular endothelial cell permeability (139). In addition, matrix metalloproteinase-9 (MMP-9) in NETs can stimulate MMP-2 in ECs, resulting in endothelial dysfunction (140). The formation and development of thrombus in sepsis are significantly associated with NETs which can intensify endothelial dysfunction.

Sepsis may induce the activation of the coagulation system, which is a crucial innate immune response that prevents the spread of microorganisms. And the thrombogenic mechanisms of NETs are diverse. NETs can trigger exogenous coagulation pathways by producing and releasing tissue factors (141) and stimulate endogenous coagulation pathways by activating coagulation factor XII (142). Neutrophils congregate and cling to ECs firmly during sepsis. Therefore, NETs are released by neutrophils and serve as a thrombosis scaffold (143). And many of these components trigger coagulation factors, such as physiological coagulation inhibitors like NE and cathepsin G, strong coagulation initiators like chromatin and histones (130, 138). Moreover, activated platelets cooperate with neutrophils to form NETs and promote thrombosis (142, 144). The formation of thrombus leads to microvascular obstruction, causing tissue ischemia and damage, which leads to multi-organ failure and death.

NETs not only cause tissue damage by inducing EC dysfunction and coagulation disorders, but they can also act as DAMP molecules, triggering or amplifying inflammatory responses directly (136). NETs can trigger the release of cytokines (IL-1β) from macrophages, thus triggering the recruitment of immune cells to promote inflammation (145, 146). In the early acute phase of ALI, NETs are essential for neutrophils interacting with macrophages, and they increase ARDS inflammatory response by encouraging macrophage polarization toward the M1 phenotype (147). In addition, NET-derived HMGB1 causes caspase-1 activation and later macrophage pyroptosis through receptor for advanced glycation end products (RAGE) and dynamic-dependent signaling, in which histones play a key role (148), augmenting inflammatory responses following sepsis. Similarly, LPS-induced NETs can induce pyroptosis of alveolar macrophages to promote the development of ARDS, and degradation of NET DNA or silencing of absent in melanoma 2 (AIM2) gene can inhibit pyroptosis of alveolar macrophages (149). These findings reveal that NETs can exert pro-inflammatory effects by influencing the release of macrophage inflammatory factors, thereby affecting the progression of inflammation after infection.

In recent years, the role of GSDMD in neutrophil NETs release has been revealed. GSDMD-mediated NET release may be triggered by gram-negative bacteria or intracellular LPS that promote inflammatory assembly and caspase cleavage (106, 110). Cleavage of GSDMD by caspase-4/5 in humans or caspase-11 in mice targets pore formation at the neutrophil granule membrane and releases NE and myeloperoxidase early in NET formation. The emission of these granule proteins triggers series of problems such as nuclear mini-cracking, DNA amplification, chromatin condensation, histone degradation and increased nuclear membrane permeability (106). Interestingly, NE initially activated GSDMD, but N-GSDMD facilitated the release of NE through the formation of granular pores, and this interaction resulted in a surge of NE concentration in the cytoplasm and further re-cleavage of GSDMD (106). The late stage of NET release also requires GSDMD, being in charge of producing pores in cell membrane to allow DNA extrusion, which is relevant to the detrimental events in sepsis, such as systemic inflammatory process and organ failure (28).

## Autophagy

Autophagy, a self-destructive process, is crucial for balancing energy sources and responding to nutritional stressors. Autophagy also performs housekeeping functions such as removing broken organelles, disordered proteins, and intracellular infections (150, 151). The process starts with a phagophore to expand to phagocytose intracellular cargo, including misfolded protein and damaged organelles, thereby isolating cargo in a double-membrane autophagosome, and then promoting autophagosomes mature by combining with lysosomes to promote degradation of autophagosomal components using lysosomal acidic proteases (150–152). A crucial regulator of autophagy, the autophagy related gene (ATG) protein is a downstream element of the mechanistic target of rapamycin complex 1 (mTORC1). ATG protein is recruited and triggered by autophagy-related stimuli to begin the production of autophagosomes (153).

**Figure 6:**
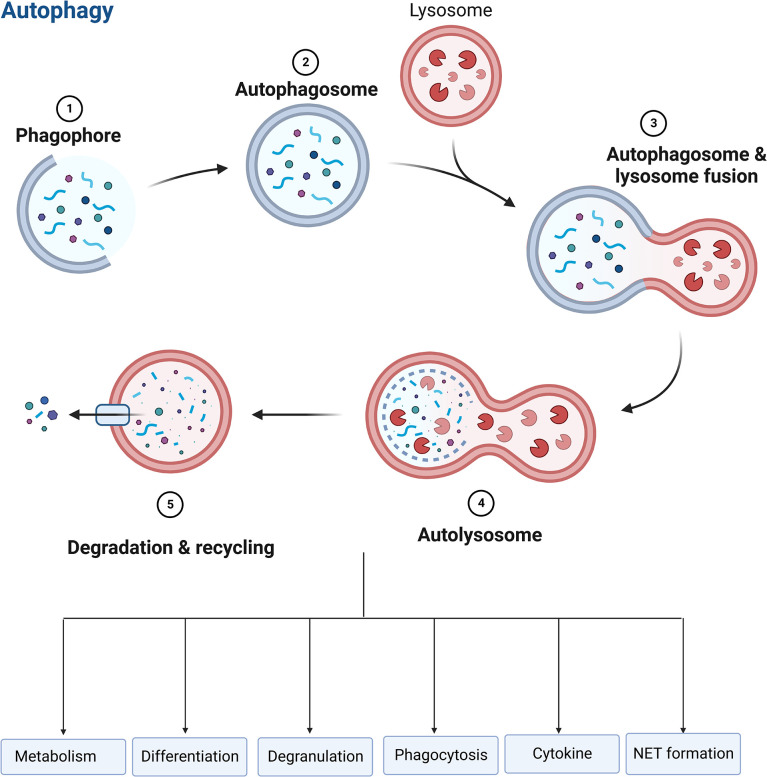
Role of autophagy in neutrophils. Autophagy is essential for regulating neutrophil function, such as metabolism, differentiation, degranulation, phagocytosis, cytokine production, and NET formation.

Autophagy is essential for regulating neutrophil function, such as metabolism, differentiation, degranulation, phagocytosis, cytokine production, and NET formation (**Figure 6**), which determine the fate of neutrophils (151, 153, 154). Autophagy offers a crucial source of energy for oxidative metabolism of neutrophil differentiation and maturation *via* mobilizing intracellular lipid reserves, which remains required to facilitate the considerable cytoplasmic and nuclear remodeling that takes place in this phase (155). It has been established that ATG5 contributes to neutrophil differentiation. ATG5 deletion will hasten neutrophil differentiation and boost bone marrow-based neutrophil precursor cell proliferation (156). And pharmacological suppression of mTORC1- or p38-MAPK-induced autophagy in neutrophilic precursor cells prevents their differentiation (156). Neutrophil degranulation and NADPH-oxidase-mediated ROS generation, which are necessary for neutrophil inflammatory activity, can be regulated by autophagy (151). The neutrophil precursors lacking ATG7 are not capable of mitochondrial respiration and exhibit excessive glycolysis, but due to impaired mitochondrial respiration, ATP production is reduced and lipid droplets accumulate (153). TLR signaling can induce macrophages autophagy as a way to get rid of a non-cognate intracellular pathogen (157). In response to TLR ligands, neutrophils also undergo autophagy. Autophagy has been linked to both phagocytosis-independent and phagocytosis-dependent initiation in neutrophils (158). Autophagy increased phagocytosis while inhibiting killing (159). In neutrophils, an autophagy-mediated secretory pathway is used to secrete IL-1β (160) and NLRP3 plays a vital role in controlling autophagy and phagocytosis (161). The extent to which autophagy plays a role in the development of NETs is still a point of contention (120, 123). Autophagy and NET induction are linked to the PI3K–AKT–mTOR axis, and mTOR inhibition enhances NET synthesis *via* autophagy. NET formation is prevented by inhibiting the PI3K type III signaling pathway, which is needed for forming autophagosome, suggesting a function for autophagy in NET formation (34, 162). Nevertheless, genetic knockout of ATG5 is associated with deficiency in autophagy, but does not cause defects in extracellular trap formation in neutrophils (35), suggesting that autophagy is not necessarily required for NET formation.

Currently, the bulk of studies on the association between autophagy and sepsis are being conducted, and many data shows that autophagy has a more beneficial effect. Septic neutrophils show increased levels of autophagy and NET formation, which improves survival in a murine sepsis model (32). A recent prospective clinical study showed that the autophagy pathway may play a significant role in the close association between increased NET formation in neutrophils and the incidence of disseminated intravascular coagulation (DIC) in patients with sepsis (163). Deficiency of ATG7 leads to increased IL-1β production and cellular scorching, resulting in impaired pathogen clearance, reduced survival and aggrevated lung injury in a murine model of *Pseudomonas* sepsis (164). Enhancing autophagy may contribute to the protective effect in organs during sepsis (165, 166). Some studies showed that activated autophagy could ameliorate inflammation, lung injury (167–169), kidney injury (170), and cardiac injury (171) induced by sepsis. In contrast, a few studies demonstrated an opposite conclusion. The increase in plasma mtDNA and protein is another characteristic of sepsis, suggesting that damaged mitochondria are not only eliminated intracellularly, but also extruded through the exocytosis of autolysosomes, which provokes an inflammatory response of immune cells (172).

## Neutrophil cell death and potential therapy for sepsis

The various types of neutrophil cell death during sepsis undoubtedly have a significant influence on how the condition progresses. According to the above summary, one of our aims is to figure out how to govern the progression of neutrophil cell death toward the protection of the body. Despite the paucity of research in this field, there are still some studies that may provide some reference for future therapeutic strategies (**Figure 1**).

Delayed apoptosis of neutrophils in sepsis is a major causative factor in septic organ dysfunction. Then how to accelerate the apoptosis of septic neutrophils may be an effective treatment for this disease. We previously found that knockdown of neutrophil PD-L1 reversed the apoptotic delay and ameliorated sepsis-induced lung injury (10). In addition, anti-PD-L1 antibody application improved survival in septic mice (173). These studies suggest that anti-PD-L1 treatment may be an effective target for the treatment of sepsis by promoting apoptosis in septic neutrophils. In 2017, Dorward et al. found that cyclin-dependent kinase (CDK) inhibition with AT7519 triggered sepsis-inducing ARDS neutrophil apoptosis, which was viewed as the first pharmacological compound to reverse delayed neutrophil apoptosis in ARDS (174). In 2019, Zhang et al. reported that nanoparticles that selectively target inflammatory neutrophils could induce neutrophil apoptosis and increase survival in patients with sepsis (77). These studies on induction of apoptosis in septic neutrophils provide new insights for the treatment of sepsis.

With the continuous research on the mechanism of pyroptosis, pyroptosis is considered to be one of the important forms of neutrophil function, but the potential value of neutrophil pyroptosis in the treatment of sepsis has not received sufficient attention. It has been suggested that regulation of neutrophil pyroptosis may have a positive impact on the therapeutic treatment of sepsis (103). Numerous research had demonstrated that inhibiting Caspase-1/11 and IL-1β/IL-18 increases survival in animal models of CLP (112, 175–177), but clinical trials have been unsuccessful (178). In 2021, there were new developments in disulfiram, an FDA-approved targeted drug for NETs and pyroptosis, which inhibited septic neutrophil GSDMD activation and reduced NET release, thereby improving sepsis-induced organ damage and survival in septic mice (28). GSDMD appears to be a promising target for the treatment of sepsis by connecting pyroptosis and NET.

Another potential treatment strategy for sepsis is anti-NETosis and neutralization of DAMPs brought about by NET release. Some studies have shown that inhibition of NETosis with PAD4 inhibitors or degradation of NETs with DNase 1 improved survival in sepsis models (179), but other reports have yielded the opposite effect (180, 181). According to Mai et al., treating DNase 1 may attenuate organ damage and improve prognosis in a sepsis model (182). NETs contain DAMPs such as histones, DNAs. Anti-histone therapy was first proposed in 2009 (138). Heparin or anti-histone antibodies have been shown in later investigations to neutralize extracellular histones and lessen their toxicity, increasing survival in animal models of sepsis, but no clinical studies have been reported (138, 183–186). Non-anticoagulant heparin is more promising than traditional heparin in this sense for potential clinical uses in the future (186). Additionally demonstrated to increase survival in animal models of sepsis is the administration of DNase 1 for digesting free DNA (187). Development of drugs targeting NETs and DAMPs appears to be a promising therapeutic target for sepsis.

## Conclusion

The pathophysiology of sepsis is heavily influenced by neutrophil cell death, but the type of cell death and the mechanisms involved are not well understood. Apoptosis has been the major focus of research on neutrophils in sepsis, but necrosis, necroptosis, pyroptosis, NETosis, and autophagy may also play an important role in this critical situation (9). At present, it is well known that neutrophil apoptosis is inhibited, NET release is increased and autophagy level is increased during sepsis (**Table 1**). However, the other types of cell death in neutrophils remains to be clarified in sepsis. Now, there is controversy about NETosis, which was once thought to be a unique type of cell death in neutrophils, and it is now thought that the release of NET may not be necessarily accompanied by cell death. Neutrophils are thought to have the ability to resist caspase-1-mediated pyroptosis (105, 106, 188), but the GSDMD can be cleaved by caspase4/5/11 to induce pyroptosis. GSDMD is believed to participate in pyroptosis and NET-mediated cell death pathways (**Figure 1**) (40), which implies that the interaction between NET formation and pyroptosis will also be an interesting topic for further researches.

Neutrophil cell death may be dysregulated during sepsis, and not all cell death types serve as positive host protection. Necrosis, necroptosis, NETosis, pyroptosis may consume neutrophils, but more importantly, they will induce exacerbation of inflammation. It is critical for neutrophils to maintain their viability during sepsis to engulf invading microorganisms and kill pathogens extracellularly through programmed release of DNA and granule proteins. In fact, neutrophil cell death in sepsis may coexist in multiple forms, which is particularly important for the regulation of host status (**Figure 1**). The distribution of neutrophil cell death may be determined by the expression of specific virulence factors by various pathogens in sepsis, the duration and intensity of stimulation, the degree of tissue damage and the response of the immune system.

Although the mechanism of neutrophil cell death in sepsis remains to be investigated, and the drug intervention targeting at neutrophil death is even rare, there is evidence that neutrophils with dysregulated death types are more harmful than protective (132). Targeting at neutrophil cell death may be a promising treatment against sepsis. Firstly, we need to determine how neutrophils die and how neutrophil cell death induces organ dysfunction during sepsis. Secondly, it would be interesting to clarify the central pathways leading to the disparity of neutrophil cell death in different timepoints and locations in septic patients. Finally, how to enlarge the protective effect and reduce the harmful effect of neutrophil cell death is a promising direction worthy of exploration in the future, and when to intervene neutrophil death also needs to be clarified.

## Author contributions

C-LZ and J-FW reviewed the literature, wrote drafts of the manuscript and prepared figures. YW, X-MD, QL, H-RL, C-MY, and PL helped in evaluation of the literature and submit manuscript. J-FW and X-MD designed and supervised the work. All authors contributed to the review and approved the submitted version.

## Funding

This work was supported by National Natural Science Foundation of China (No. 82072147), Shanghai Rising-Star Program (21QA1411800), Sci-Tech Innovation 2030 Brain Science and Brain-Like Intelligence Technology Project (2022ZD0208100).

## Acknowledgments

Figures in this review are made by BioRender (https://app.biorender.com/).

## Conflict of interest

The authors declare that the research was conducted in the absence of any commercial or financial relationships that could be construed as a potential conflict of interest.

## Publisher’s note

All claims expressed in this article are solely those of the authors and do not necessarily represent those of their affiliated organizations, or those of the publisher, the editors and the reviewers. Any product that may be evaluated in this article, or claim that may be made by its manufacturer, is not guaranteed or endorsed by the publisher.
